# Functional Pulmonary Imaging

**DOI:** 10.1002/jmri.29778

**Published:** 2025-04-11

**Authors:** Agilo L. Kern, Filip Klimeš, Andreas Voskrebenzev, Hoen‐Oh Shin, Jens Vogel‐Claussen

**Affiliations:** ^1^ Institute for Diagnostic and Interventional Radiology Hannover Medical School Hannover Germany; ^2^ Biomedical Research in Endstage and Obstructive Lung Disease Hannover (BREATH) German Center for Lung Research (DZL) Hannover Germany

**Keywords:** alveolar membrane function, imaging, lung function, microstructure, perfusion, ventilation

## Abstract

The aging of the world population gave rise to an increased prevalence of many lung diseases, with chronic obstructive pulmonary disease now ranking as the third‐leading cause of death according to the World Health Organization. To diagnose lung disease, a thorough assessment of lung function is essential since it may reveal unique signatures in terms of disease pathophysiology. Yet, clinically established lung function tests are global measurements, which may compromise their sensitivity to early, regional changes in lung function compared to spatially resolved imaging tests. From a scientific perspective, the lung is a highly complex organ, and newly developed functional imaging methods may elucidate previously unknown aspects of its physiology. Functional pulmonary imaging is and will thus be of great value for both clinical and research applications. The goal of this review is to shed light on the field of functional pulmonary imaging in all its varieties, with a particular focus on the numerous tools MRI has to offer. This includes ^1^H MRI methods with or without exogenous contrast agents like oxygen‐ or gadolinium‐based contrast agents and MRI of hyperpolarized and inert gases like ^129^Xe or perfluoropropane. However, thinking outside the box, a glance is also taken at what other modalities like single‐photon emission computed tomography, computed tomography, or X‐ray dark‐field imaging have to offer. Following a physiological perspective, methods are described in terms of their ability to assess the key parameters of lung physiology in humans—ventilation, perfusion, and alveolar membrane function, as well as microstructure—and promising clinical and research applications are discussed. An outlook into possible future paths the field might take is given.

**Evidence Level:** 5.

**Technical Efficacy:** 2.

## 1 Introduction

Lung diseases pose a significant burden on the world population, with chronic obstructive pulmonary disease (COPD) being the third leading cause of death worldwide, according to figures of the World Health Organization from 2019 [1].

Lung function assessment has been of high relevance for diagnosis, monitoring, and prognostication of patients with lung disease. Compared to the use of conventional lung function testing, pulmonary functional imaging seems a young and rapidly developing field. Pulmonary functional imaging as a toolbox of noninvasive imaging methods may still elucidate novel and clinically relevant knowledge of the normal function of the lung.

The purpose of this article is to provide an overview with a focus on MRI methods but with the intention to span all modalities. This overview should be considered non‐complete given the broadness and rapidly evolving nature of the field. The article is structured according to a physiological perspective, following the different aspects of lung function.

### 1.1 Lung Physiology

Basic knowledge of lung physiology and structure has historically been obtained in great part by microscopic imaging [2] given the past and partly still present limitations of noninvasive imaging methods in terms of, for example, image resolution.

Gas exchange may be considered the primary function of the lung. To achieve this, air needs to be transported to and from the gas exchange surface efficiently. The human lung possesses an intriguing compact tree‐like structure of airways separating further and further in approximately 23 generations from the trachea to the level of the gas exchange area in alveolar ducts and sacs. Air is moved through this structure in and out, leading to lung *ventilation*. Given the increase of the sum of cross‐sectional areas of airways with increasing splitting, movement of fresh and used air within the distal individual airways becomes very slow and is ultimately dominated by passive diffusion.

The heart pumps oxygen‐depleted blood through the capillary network in the gas exchange region—the parenchyma. The volume of this network is only around ~200 mL [2]—the same order of magnitude as cardiac stroke volume, meaning that the lung parenchyma is continuously *perfused* with blood, spending a time in the range of seconds within capillaries, depending on heart rate and cardiac function. After uptake of oxygen and removal of carbon dioxide in the gas exchange area, the blood returns to the heart to enter the systemic circulation and to deliver oxygen to the organs in order to generate adenosine triphosphate (ATP), a readily available form of energy in the cells through oxidative decarboxylation.

Assuming a uniform spread of the capillary blood volume over the enormous alveolar surface area of ~10^2^ m^2^ available for gas exchange, the blood is seen to form a sheet‐like structure with a mean thickness in the range of micrometers. This is less than the length scale corresponding to the root‐mean‐square displacement in Brownian diffusion of order *√Dt* for small molecules like oxygen within solution during a time scale of the capillary transit time of ~1 s and with a diffusion coefficient ~10^3^ μm^2^/s [3]. The *alveolar membrane* separating airspaces and blood also has a thickness in the micrometer range to allow for efficient gas exchange by passive transport processes.

To achieve all this within a limited volume of the thoracic cavity and with sufficient structural stability, the lung possesses a complex, optimized *(micro‐)structure*, which is only touched upon in this article.

**FIGURE 1:**
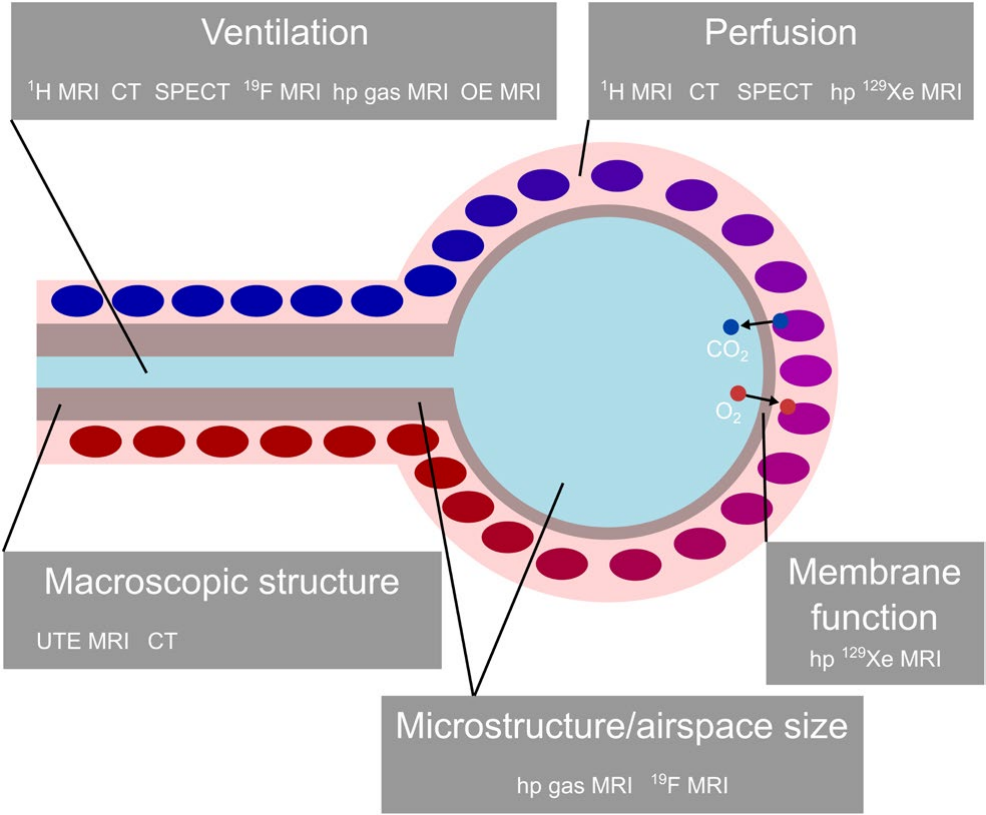
Overview of the methods available for functional lung imaging and described in this article. The methods for assessment of structure/membrane function are roughly sorted according to the length scales probed. CT, computed tomography; hp, hyperpolarized; OE, oxygen‐enhanced; SPECT, single‐photon emission computed tomography; UTE, ultra‐short echo time.

This article describes pulmonary functional imaging in the four aspects of ventilation, perfusion, alveolar membrane function, and microstructure with dedicated sections. Figure 1 shows an overview of the individual imaging modalities and methods available for assessing aspects of lung function. Table 1 summarizes the relative strengths and weaknesses of the methods.

**TABLE 1:** Comparison of imaging methods.

Physiological aspect	Method	Technique	Relative strengths	Relative weaknesses
Ventilation	Hyperpolarized gas MRI (^129^Xe, ^3^He)	Single‐breath imaging	High‐resolution, sensitivity for disease	No quantification of ventilation in physiological units
		Multibreath imaging	Absolute quantification	Low‐resolution, technical complexity, less established
	^19^F MRI	Multibreath imaging	Absolute quantification, no need for hyperpolarization hardware, no anesthetic effects	Lower resolution/SNR compared to hyperpolarized gases, potent greenhouse gases
	^1^H MRI	2D PREFUL	Technical simplicity, no additional hardware, patient‐friendly examination, simultaneous perfusion information	Computationally intensive post‐processing, indirect ventilation assessment
		3D PREFUL	Fast acquisition for whole‐lung coverage	Comparatively long scan times (~8 min), typically no perfusion information, computationally intensive post‐processing
	Oxygen‐enhanced MRI	Multibreath	Ready availability, quantitative measurement in dynamic protocols	May be technically complex, long scan times, weak signals
	SPECT	^99m^Tc‐labeled aerosol	Clinically established gold standard	Limited resolution, ionizing radiation, cost
	CT	Xenon CT	Technical simplicity	Ionizing radiation, anesthetic effect
		Acquisition in in‐/exhalation	High resolution, no tracer gas required	Increased radiation dose, indirect measurement
Perfusion	DCE MRI	First‐pass imaging after bolus injection	Established method, quantification in physiological units, high resolution, fast acquisition	Risks of gadolinium‐based contrast agents
	ASL MRI	Pseudo‐continuous ASL	Quantification in physiological units, no contrast agent required	Long acquisition times, limited lung coverage
	Fourier decomposition‐based MRI	2D PREFUL	No contrast agent required	Long acquisition times, computationally intensive post‐processing, quantification difficult
	^129^Xe MRI		Can be specific to capillary blood	Quantification in physiological units difficult, validation pending
	Contrast‐enhanced CT	Helical scanning	Fast, high resolution	No quantification in physiological units, ionizing radiation
		Dynamic axial scanning	Quantification in physiological units	High radiation dose, limited lung coverage
	SPECT		Gold standard method, low radiation dose compared to CT, simultaneous ventilation assessment	Low resolution
Alveolar membrane function	^129^Xe MRI	Dissolved‐phase imaging	Comparatively low resolution achievable	Signals may be hard to interpret, large dose associated with anesthetic effects required
		Chemical shift saturation recovery	Quantification of morphometric quantities, additional dynamic information	Typically, only global measurement, difficult to perform imaging in one breathhold
Microstructure	Diffusion‐weighted MRI of inhaled tracers (^129^Xe, ^3^He, ^19^F)	ADC mapping	High sensitivity for disease, fast measurement	Results dependent on sequence parameters
		Microstructure quantification using multiple *b*‐value experiments	Accurate quantification using established models	Models may not be representative of diseased lung tissue
		Microstructure quantification using multiple diffusion time experiments	Simple theoretical, model‐free relationship	Short enough diffusion times may be difficult to achieve
	UTE MRI		No additional hardware required	Lower resolution compared to CT, results may be hard to interpret
	CT		High resolution, ready availability	Microstructure quantification only possible in special cases
	Dark‐field X‐ray/CT imaging		Relative technical simplicity	Results may be challenging to interpret in terms of morphometry, development/validation ongoing

Abbreviations: ADC, apparent diffusion coefficient; ASL, arterial spin labeling; CT, computed tomography; DCE, dynamic contrast‐enhanced; PREFUL, phase‐resolved functional lung; SPECT, single‐photon emission computed tomography; UTE, ultra‐short echo time.

## 2 Ventilation

Adequate ventilation (abbreviated V) of the lungs is of key importance for efficient gas exchange. Ventilation is usually impaired regionally in obstructive as well as restrictive lung diseases.

In spite of their presumed high sensitivity, imaging tests typically do not play a role in the diagnosis of obstructive lung disease, which is based on simple methods like spirometry. Clinical applications where ventilation imaging can be most beneficial include treatment monitoring and assessment of disease severity, and identification of target regions for interventions like lung volume reduction surgery, endobronchial valve placement, or radiotherapy of lung cancer.

### 2.1 Hyperpolarized Gas MRI

**FIGURE 2:**
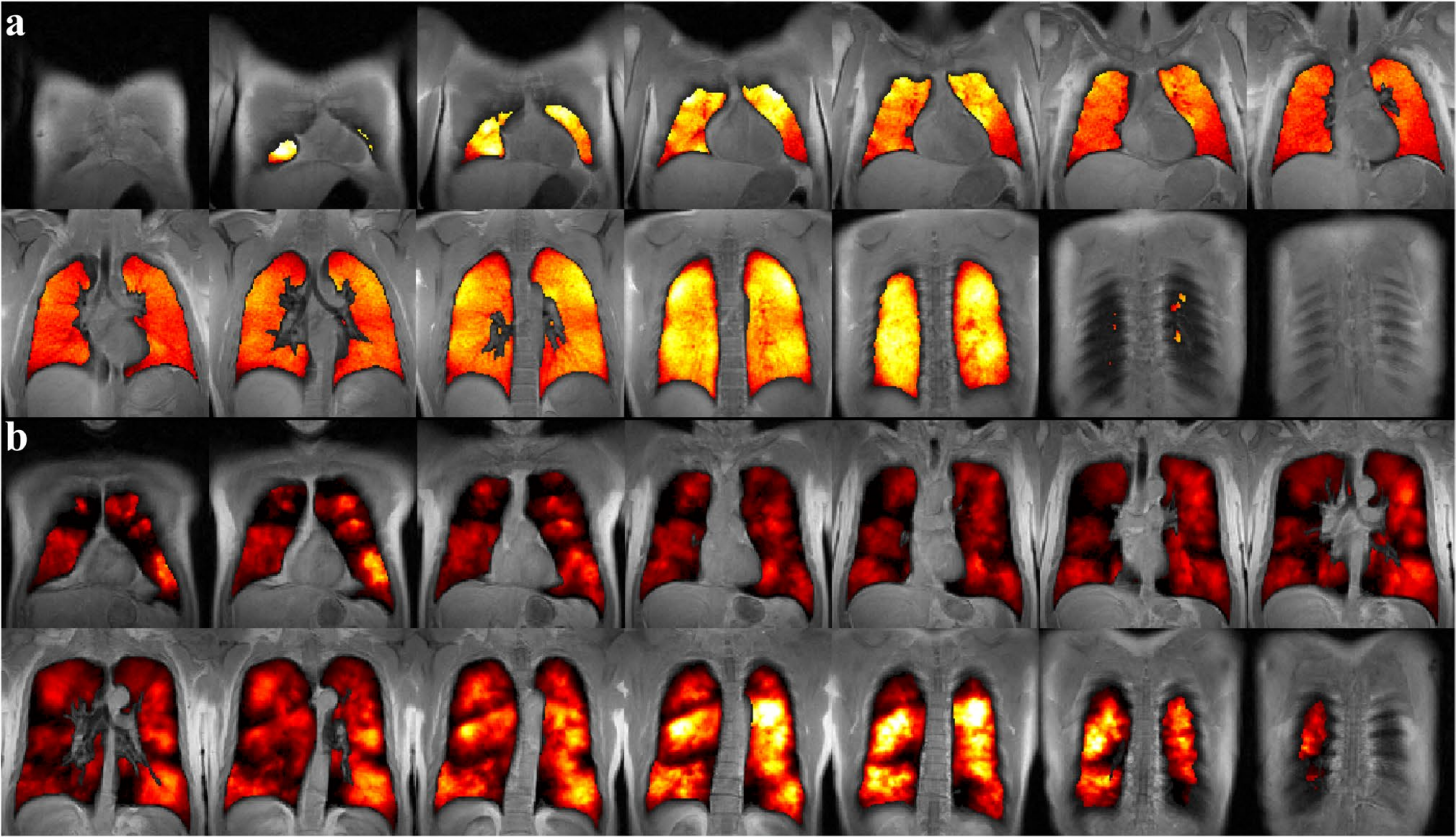
Ventilation imaging with hyperpolarized ^129^Xe in (a) a healthy volunteer (24‐year‐old male, FEV_1_ 102.2% of predicted value) and (b) a patient with chronic obstructive pulmonary disease (73‐year‐old male, FEV_1_ 55.4% of predicted value). ^129^Xe images (color) are overlaid over conventional anatomic ^1^H images (gray) within the thoracic cavity. Marked heterogeneity of ventilation is observed throughout the lung in the patient, whereas ventilation is very homogeneous in the healthy volunteer. FEV_1_, forced expiratory volume in 1 s.

Images of the distribution of hyperpolarized gases after single inhalation were found to be very useful in obstructive lung disease soon after the introduction of MRI of the spin 1/2 nuclei ^3^He and ^129^Xe in their hyperpolarized state to biomedical imaging [4, 5, 6]. These images show signals directly proportional to ventilation. Figure 2 shows representative imaging results in a patient with COPD.

Sequences are generally of the fast low‐angle shot (FLASH) or true fast imaging with steady‐state precession (TrueFISP) type [7] and high spatial resolution with isotropic voxel sizes in the range of a few millimeters can be obtained in one breathhold, even for the isotope ^129^Xe which typically provides lower SNR than ^3^He due to differences in achievable polarization levels and gyromagnetic ratios [8].

The primary parameter obtained from these images is typically the percentage of ventilation defects within the lung. Difficulties in the quantification may arise through variable and hard to determine bias fields as well as inaccurately known lung boundaries in non‐ventilated areas despite recent progress [9, 10]. The problem of misalignment between signals in gas and anatomical ^1^H images may be addressed by sequential [11] or potentially truly simultaneous acquisition at the two Larmor frequencies [12].

Numerous studies have been conducted showing the sensitivity of hyperpolarized gas ventilation MRI to diseases like COPD [13], asthma [14], or cystic fibrosis [15] and also to changes due to treatment [16, 17]. Albeit this is the most mature and only clinically applied MRI method of directly assessing ventilation using hyperpolarized gases, a limitation is that ventilation is not quantitatively assessed.

Alternatively, ventilation may be studied using an image time series. Buildup of signal during the wash‐in phase of the tracer gas may be used to calculate fractional ventilation as the relative amount of gas exchanged per breath [18]. Similar methods are applicable to the wash‐out of the gas during inhalation of, for example, room air not containing the tracer gas [19]. After initial work in preclinical settings, multiple‐breath ventilation imaging has also been applied in human studies [20]. The quantitative assessment of ventilation compared to images showing signal intensities proportional to ventilation may be considered an advantage. Disadvantages are the increased complexity of the imaging protocol, reduced spatial resolution, and more elaborate post‐processing to compensate for the effects of polarization decay. Further, given present polarization technology and the high cost of the isotopes ^3^He and ^129^Xe, the amount of gas that can be used is limited such that a complete wash‐in of gas may be difficult to achieve in slowly filling regions.

While clinical translation of hyperpolarized gas MRI has been predicted early [21], it has—so far—mostly been limited to clinical research. The recent approval of hyperpolarized ^129^Xe MRI for ventilation imaging in the United States could change this. One of the reasons for the slow translation may be the expensive equipment, with the scope of applicability limited mostly to lung imaging, thus likely restricting dissemination to specialized centers. Another reason may have been the initial focus on ^3^He due to technically easier polarization and better relaxation characteristics allowing for remote production, while this isotope is not abundant on earth in sufficient amounts. ^129^Xe naturally occurs in the atmosphere, which makes it more promising in terms of clinical translation combined with improvements in polarization technology in recent years. The large natural isotopic fraction of 26.4% of ^129^Xe facilitates clinical application without the need for costly isotopic enrichment, as is typically done for research studies [22]. Use of a lower isotopic ratio may require a higher volume of xenon to compensate for the loss in signal‐to‐noise ratio, however, which may lead to increased anesthetic side effects observed at higher volumes around 1 L [23] and patient discomfort.

### 2.2 ^19^F MRI

The idea of using the spin 1/2 isotope ^19^F with its high gyromagnetic ratio of ~2π × 40 MHz/T and 100% natural abundance for lung imaging is relatively old [24]. Compared to ^129^Xe and ^3^He which are almost MR‐invisible in the thermally polarized state due to long spin–lattice relaxation times, fluorinated gases like perfluoropropane can be used for imaging at thermal polarization due to the very short spin–lattice relaxation times enabling rapid signal averaging. The use of fluorinated gases is associated with reduced regulatory hurdles compared to hyperpolarized gases since there is no need for local gas production. Images also do not suffer from the detrimental effects of polarization decay, which are not always easy to quantify. Another advantage is the ability to use large volumes of gas.

The later aspect has proven to be very beneficial for studying wash‐in and wash‐out dynamics in multiple‐breath protocols. The delayed wash‐out of fluorinated gases has been shown to be sensitive to COPD and to correlate with the severity of airway obstruction as seen in spirometry [25, 26]. Monitoring of cystic fibrosis patients may be an application where MRI can play a key role in general due to lack of ionizing radiation, and it was demonstrated that multiple‐breath wash‐out MRI using fluorinated gases is sensitive to cystic fibrosis in patients with normal forced expiratory volume in 1 s [27].

Despite these promising results, the method has so far been adopted by only a few research sites worldwide. Images after single inhalation may be sensitive to mild obstruction but suffer from low SNR. Also, the need for dedicated RF hardware may still hinder widespread clinical dissemination. Further, the imaging properties of fluorinated gases reduce versatility compared to imaging of hyperpolarized gases and limit achievable spatial resolution despite recent improvements through advanced reconstruction [28]. There may also still be room for improvement in terms of the optimal choice of the imaging agent [29].

### 2.3 ^1^H MRI

A promising MRI method for indirect ventilation assessment utilizing conventional ^1^H imaging is to continuously acquire a 2D slice during free breathing without contrast agent application. Ventilation information is obtained after image registration from the respiration‐induced variation of the lung parenchyma's signal intensity in the image stack [30]. Ventilation is quantified using a sponge model, assuming a linear relationship between signal intensity and tissue density [31].

**FIGURE 3:**
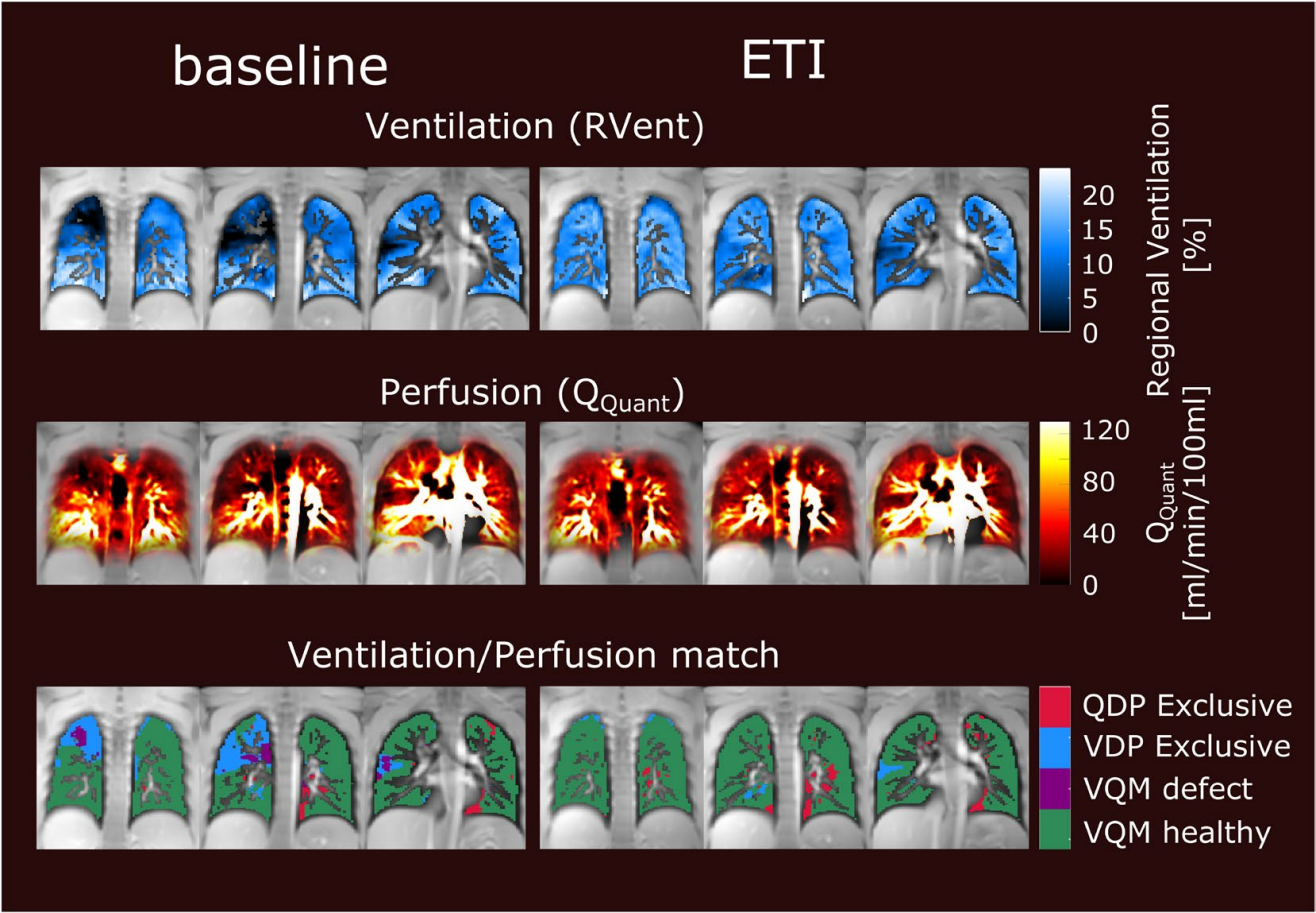
2D PREFUL ventilation maps (first row), perfusion maps (second row), and ventilation/perfusion match classification (third row) in an 18‐year‐old male cystic fibrosis patient before and 13 weeks after beginning treatment with triple combination therapy elexacaftor/tezacaftor/ivacaftor (ETI). PREFUL, phase‐resolved functional lung; QDP, perfusion defect percentage; QQuant, quantified perfusion; RVent, regional ventilation; VDP, ventilation defect percentage; VQM, ventilation/perfusion match.

Different post‐processing approaches may be used to extract the ventilation‐weighted information from the signal time series, including spectral analysis with Fourier decomposition, low‐pass filtering with subsequent ventilation phase sorting (phase‐resolved functional lung MRI (PREFUL) [32]) or sorting prior to reconstruction (self‐gated noncontrast‐enhanced functional lung MRI (SENCEFUL) [33]). Phase sorting offers the advantage of facilitating the calculation of regional ventilation flow volume loops, comparable to spirometric measurements. Several studies suggest that this dynamic information may offer increased sensitivity over simply comparing end‐inspiratory and end‐expiratory states. This is particularly evident in applications such as predicting graft loss following lung transplantation [34] or monitoring treatment outcomes in COPD [35]. See Figure 3 for representative PREFUL ventilation maps applied for monitoring treatment in cystic fibrosis. Despite the indirect assessment of ventilation, relatively good agreement of ventilation defect percentages with results from, for example, ^129^Xe MRI was found in several diseases [36, 37].

While the technical simplicity during image acquisition has proven to be highly beneficial for clinical dissemination, the complex image post‐processing and associated computational requirements remain challenging. Most time‐consuming is non‐rigid registration of the images. Probably due to low signal‐to‐noise ratio in the lung region, averaging images of comparable respiration states was shown to be beneficial [38]. However, great improvements in registration speed have recently been achieved [39] and more improvements in the coming years are likely, for example, by the use of artificial intelligence‐based methods or GPU‐accelerated registration strategies.

A potential clinical application of signal‐based ^1^H ventilation MRI is monitoring of, for example, cystic fibrosis patients in treatment for pulmonary exacerbations [40]. In addition, infants and neonates and potentially preterm babies may be imaged [41]. Other patient groups requiring dense follow‐up imaging, like lung transplant recipients, could benefit from this method, too [42]. Signal‐based ^1^H ventilation MRI methods have also been applied and compared to hyperpolarized gas imaging in COPD, asthma, and bronchiectasis, and it was suggested that agreement may vary between diseases [43, 44].

Another method proposed for obtaining ventilation information from dynamic ^1^H MRI is to use the Jacobian determinant describing the local volume changes of lung tissue [45]. One of the advantages may be the fact that changes in transverse relaxation with lung inflation level do not negatively affect the results [46]. Due to constraints on the deformation field imposed by image registration algorithms, the ventilation images obtained may have reduced effective spatial resolution compared to signal‐based methods. Studies of lung physiology with respect to, for example, gravitational gradients may be an application for which the method could be well suited. Results of such Jacobian determinants have been reported in healthy volunteers and patients with lung fibrosis [47]. Yet, a recent validation study using a synthetic lung model showed impaired reliability compared to signal‐based methods [48].

**FIGURE 4:**
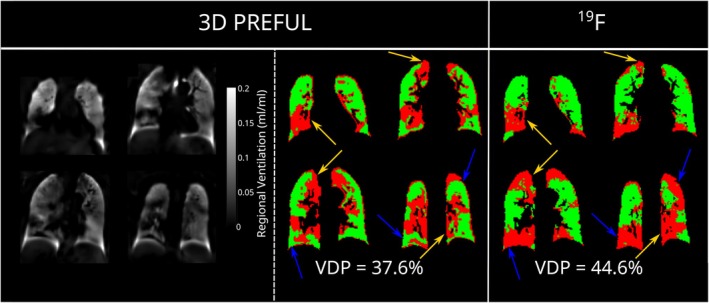
3D PREFUL MRI regional ventilation maps (on the left), respective ventilation defect maps (in the middle), and ^19^F (on the right) ventilation defect maps for a 60‐year‐old male with chronic obstructive pulmonary disease (FEV_1_ 53% of predicted value). Defect regions are color‐coded in red, gold arrows show the regional ventilation defect agreement, and blue arrows point out the regional differences. Sørensen–Dice coefficient was 61.8% and 48.2% for healthy and defect areas, respectively. FEV_1_, forced expiratory volume in 1 s; PREFUL, phase‐resolved functional lung.

Among the general technical limitations of 2D acquisitions in ^1^H MRI for ventilation assessment are the relatively long scan times necessary for obtaining ventilation maps with whole‐lung coverage. Through‐plane motion of lung tissue may negatively affect the results and impose restrictions on slice orientations. A possible solution is a 3D acquisition. Ventilation information may be obtained similarly as in the 2D case with a subsequent analysis of signal variation after registration or of deformation fields in the lung parenchyma within about 8 min [33, 49, 50]. The 3D PREFUL technique has been demonstrated to be feasible for ventilation assessment in patients with obstructive lung disease, and good global agreement with results from fluorinated gas and hyperpolarized ^129^Xe MRI has been found [51, 52, 53]; see Figure 4. Among the disadvantages of this method is the loss of perfusion information readily obtainable in 2D ^1^H MRI acquisitions, as will be described in more detail below.

### 2.4 Oxygen‐Enhanced MRI

By virtue of its paramagnetic properties, molecular oxygen dissolved in lung tissue and blood may influence the MR signal in ^1^H MRI through the increase in spin–lattice relaxation rate R_1_. It thereby acts as a natural contrast agent. Measurement of R_1_ rates/T_1_ times under inhalation of gases with varying fractions of oxygen may be used to study oxygen uptake through the oxygen transfer function defined as the slope of R_1_ as a function of oxygen fraction [54]. In addition, oxygen inhalation may influence the blood oxygenation level, leading to changes in apparent transverse relaxation times (BOLD effect). Both T_1_ and T_2_* weighted imaging may thus be sensitive to signal changes upon oxygen inhalation.

Previous studies have shown, for example, the potential of oxygen‐enhanced MRI to detect pulmonary functional loss in patients with COPD and to discriminate patients with varying disease severity [55]. A great advantage of oxygen‐enhanced MRI is the fact that both technical and regulatory hurdles are low since it relies on ^1^H MRI contrasts and medical oxygen is readily available, which could make it interesting for clinical application. The depiction of the uptake of oxygen may be particularly physiologically relevant as its extraction is one of the primary functions of the lung. However, a limitation of static oxygen‐enhanced MRI is the fact that the biomarkers obtained are likely reflective of a combination of ventilation, perfusion, and diffusion through the blood–air barrier, and for this reason, they are not always easy to interpret in terms of physiology, which may hamper clinical interpretation.

This limitation may be addressed by dynamic oxygen‐enhanced MRI protocols. By assuming a linear relationship between oxygen concentration in lung tissue and R_1_ relaxation rates, quantification of the correlation delay time between MRI signals and switching inspired oxygen fraction over a multitude of breaths may be used to accurately quantify specific ventilation, for example, to elucidate aspects of normal lung physiology [56]. The long scan times necessary for quantification in 2D slices seem problematic with respect to clinical implementation, which could potentially be addressed by use of 3D imaging methods [57].

### 2.5 SPECT

Ventilation imaging using single‐photon emission computed tomography (SPECT) may be achieved by inhalation of a radioactive gas or a labeled radioactive aerosol emitting gamma rays. In the case of an aerosol, the distribution of inhaled particles within the lung, in particular penetration to the lung periphery, is dependent on particle size. The results obtained hence depend on the radioactive aerosol used and differences appear to be particularly pronounced in obstructive lung disease, for which Technegas is recommended [58]. Regional ventilation deficits, for example, in COPD, can be detected [59]. Disadvantages are the comparatively low spatial and temporal resolution, use of ionizing radiation limiting use for follow‐up imaging, and expensive radioactive isotopes with limited availability.

### 2.6 CT

Computed tomography (CT) may be used to obtain information on regional ventilation, and a wide variety of methods exist. For example, dual‐energy CT after inhalation of a gas with a high atomic number like xenon may be used to estimate the component of attenuation caused by xenon and consequently local ventilation [60]. While the ventilation images produced are of high quality, one downside for the clinical application, for example, in treatment monitoring, is the exposure to ionizing radiation. Protocols with prolonged inhalation of xenon may also be associated with increased anesthetic effects [60].

**FIGURE 5:**
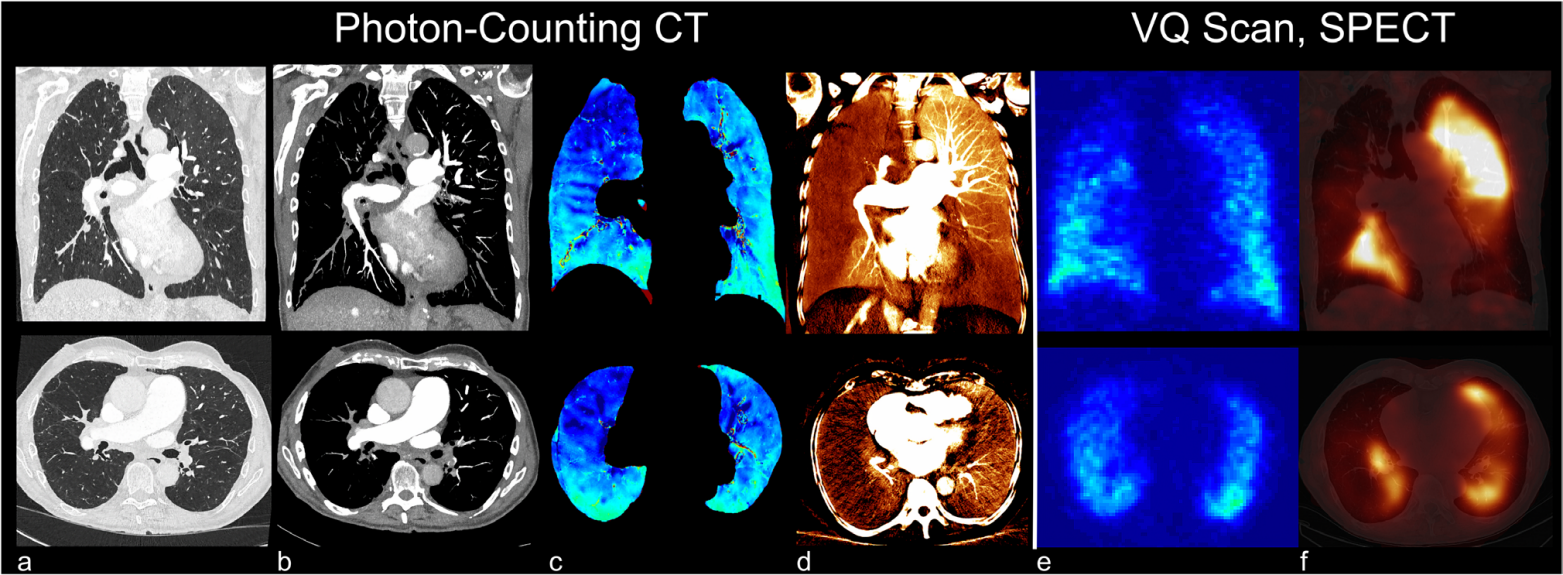
Photon‐counting CT images of a patient with chronic thromboembolic hypertension (CTEPH). Photon‐counting CT allows a comprehensive assessment of the lung structure and vasculature (a, b) and function (ventilation and perfusion: c, d) with high spatial resolution. CT ventilation and perfusion (c, d) show high agreement with VQ‐SPECT (e, f). VQ‐SPECT, ventilation/perfusion single‐photon emission computed tomography.

Other methods are based on acquiring images at inspiration or expiration, at multiple lung inflation levels, or even dynamically with subsequent deformable image registration. Ventilation may then be derived from the change in CT density in Hounsfield units or from the Jacobian determinant of the deformation field [61]. A somewhat different approach is taken by parametric response mapping, which classifies density changes with inflation level into normal lung, functional small airways disease, and emphysema to study obstructive lung disease [62]. Similar to ^1^H MRI methods, while technically simple during the image acquisition phase, more work is required on the post‐processing side. Repeated or prolonged measurements may further lead to a high radiation dose. However, recent advances with very promising clinical potential have been made using photon‐counting CT technology, which allowed for a dose‐efficient and robust simultaneous evaluation of pulmonary morphologic structure, ventilation, vasculature, and parenchymal perfusion in a procedure requiring advanced software but no additional hardware [63], see Figure 5.

## 3 Perfusion

Pulmonary perfusion (abbreviated Q) is altered in a wide range of lung diseases, for example, by the Euler–Liljestrand mechanism or other factors like pulmonary embolism. Although contrast‐enhanced CT and nuclear medicine methods like SPECT are more established for the clinical assessment of pulmonary perfusion defects [59, 64], several MRI methods exist for the assessment of different aspects of pulmonary perfusion from macro‐ to microscopic scale.

### 3.1 Dynamic Contrast‐Enhanced MRI

Dynamic contrast‐enhanced (DCE) MRI is the most established MRI technique for assessment of lung perfusion. The most established method is to image the first pass of a single bolus of gadolinium‐based contrast agent after intravenous injection. Besides perfusion defect percentage at maximum enhancement, quantitative information on perfusion like pulmonary blood flow may be obtained from the signal evolution in T_1_‐weighted sequences using established methods [65]. This requires knowledge about the arterial inflow of contrast agent in time and making some assumptions, among them being a linear relationship between concentration of the contrast agent and signal intensity. This is frequently a relatively crude approximation for the doses necessary to achieve sufficient SNR, however. Deviations from linearity lead to a distortion of the estimated arterial input function and consequently biased results. A prior injection of contrast agent at reduced dose for estimation of the arterial input function may be used for mitigating this problem [66] but increases the complexity of the acquisition.

Among the advantages of DCE MRI for lung perfusion assessment are the comparatively high spatial resolution with full lung coverage in a single breathhold and the relatively simple acquisition and analysis. Reports of retention of contrast agents containing gadolinium in brain tissue have raised health concerns in the past [67]. However, with the introduction of more stable cyclic gadolinium‐based contrast agents, gadolinium deposition in the body and related nephrogenic systemic fibrosis has been less frequent [68]. There are also environmental concerns associated with the use of gadolinium during MRI examinations due to its accumulation in rivers and drinking water [69]. Other compounds, for example, hyperpolarized MRI tracers, are possible alternatives for first‐pass lung perfusion imaging [70].

In COPD, DCE‐MRI‐derived pulmonary microvascular blood flow (PMBF) was shown to be reduced in mild COPD, including in regions of lung without frank emphysema [71]. Also, pharmacologic intervention with inhalers improved DCE‐MRI‐derived PMBF in COPD patients with hyperinflated lungs [72]. Therefore, PMBF may provide an imaging biomarker for therapeutic strategies targeting the pulmonary microvasculature [73].

**FIGURE 6:**
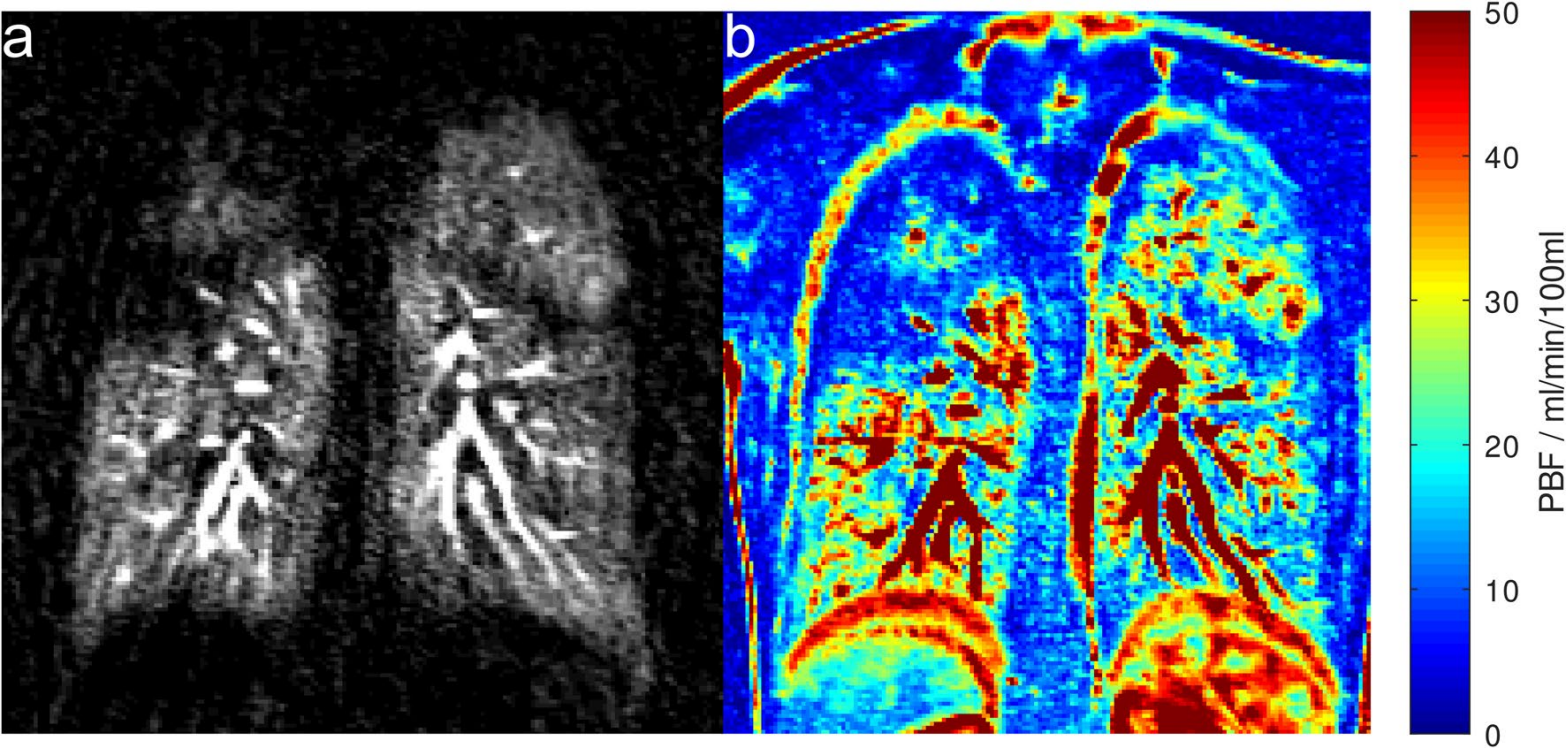
(a) Maximum enhancement in a series of first‐pass dynamic contrast‐enhanced MR images as well as pulmonary blood flow map (b) in a pediatric cystic fibrosis patient (17‐year‐old female, FEV_1_ 100% of predicted value). Perfusion defects are readily apparent mainly in the right lung. FEV_1_, forced expiratory volume in 1 s; PBF, pulmonary blood flow.

The monitoring of cystic fibrosis lung disease is an example where MRI—and DCE lung MRI in particular—may play a role in clinical practice due to the lack of ionizing radiation and the inherent information on ventilation through the Euler–Liljestrand mechanism [74]. Figure 6 shows imaging results in a pediatric CF patient. It was further demonstrated that DCE MRI has very high sensitivity for the detection of chronic thromboembolic pulmonary hypertension [75]. DCE MRI could also be of interest in the detection of acute pulmonary embolism, although a previous study showed a large number of scans with inconclusive results limiting use in an emergency setting [76].

### 3.2 ASL MRI

Arterial spin labeling (ASL) in MRI is a method for perfusion assessment using endogenous contrast created by manipulating the magnetization of inflowing blood, thus not requiring the injection of substances. In essence, an ASL acquisition consists of a labeling part and an acquisition part. In the labeling part of the sequence, water protons in a slab containing large arterial vessels are labeled by RF pulses, which later reduce the MR signal in the rapidly imaged acquisition plane through perfusion. Several variants of ASL exist in terms of labeling technique addressing issues like the absorption of RF energy or off‐resonant excitation of macromolecules in the imaging plane, reducing the signal through magnetization transfer unrelated to perfusion.

Perfusion may be quantified in physiologically meaningful units using established theories [77]. Application of ASL to study lung perfusion is viable but suffers from multiple issues, among them being the complex anatomy of vasculature within the thorax, flow within the imaging plane, flow pulsatility and low signal‐to‐noise ratio, motion, and relatively long acquisition times already for single slices [78]. These factors pose limitations on the location and orientation of imaging slices.

Clinical applications of ASL for studying lung perfusion have so far been limited, although there seems to be potential, for example, for detection of pulmonary embolism [79] or monitoring of pediatric cystic fibrosis patients [80]. Nevertheless, the technique proved to be very useful for studies of human lung physiology [81].

### 3.3 Fourier Decomposition‐Based MRI

In analogy to ventilation, Fourier decomposition and phase‐sorting methods measuring periodic changes of signal intensity may also be used for assessment of lung perfusion, at least in the case of 2D acquisitions. The perfusion contrast here relies mostly on the inflow of unsaturated spins into the imaging plane. While sharing some limitations with ASL and other approaches of perfusion weighting using ECG‐gated acquisitions [82], the technical simplicity of image acquisition using gradient‐echo sequences is advantageous in terms of clinical dissemination. A further advantage over DCE and ASL is the combined assessment of ventilation and perfusion, providing information on ventilation/perfusion (V/Q) mismatch which may be more clinically relevant than perfusion deficits alone. See Figure 3 for representative perfusion maps and ventilation/perfusion match classification in cystic fibrosis. The usual free‐breathing protocols help reduce patient discomfort although free‐breathing protocols may also be feasible for DCE or ASL [83, 84]. Good agreement of visual scores of lung perfusion from Fourier decomposition with those from DCE has been demonstrated [85]. Absolute quantification of perfusion remains challenging despite recent progress [86].

Using phase‐resolved functional lung (PREFUL) MRI, the randomly acquired images are sorted to reconstruct a whole cardiac cycle with higher apparent temporal resolution of approximately 80 ms. This enables visualization and quantification of the pulse wave transit in the pulmonary arterial and venous system and quantification of the pulse wave velocity [87, 88, 89]. These novel markers are currently under investigation for monitoring and prognostication in patients with chronic lung disease.

The potential clinical applications of Fourier decomposition‐based MRI methods for studying lung perfusion are numerous. The longitudinal follow‐up in young patients with cystic fibrosis may be an important application given the concerns of potential long‐term consequences associated with repeated gadolinium injections [90]. Lung perfusion imaging in infants [41] with, for example, bronchopulmonary dysplasia or the potentially detection of pulmonary embolism in pregnant women are other clinically highly relevant examples.

### 3.4 ^129^Xe MRI

**FIGURE 7:**
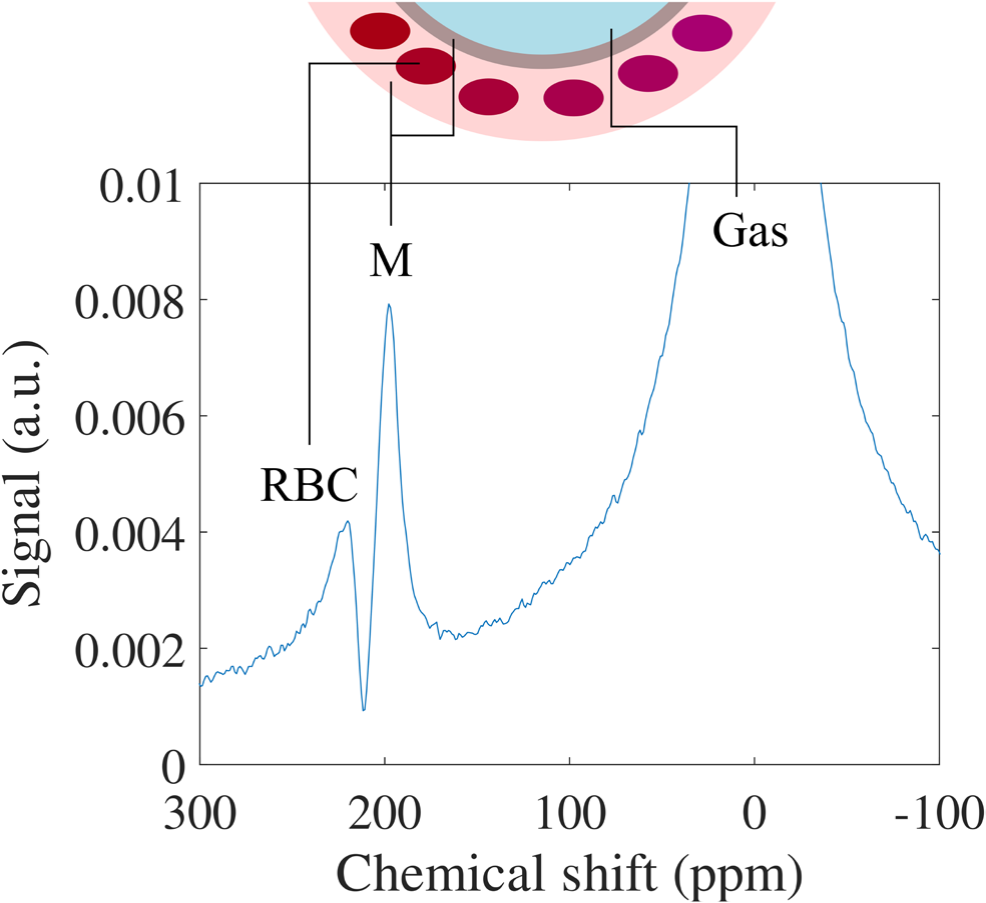
MR spectroscopy reveals that besides the strong resonance associated with gaseous ^129^Xe, there are two smaller resonances associated with ^129^Xe in membrane tissues (M) and ^129^Xe in red blood cells (RBCs). This allows the selective probing of the individual compartments in the gas uptake process.

By virtue of its good solubility and large chemical shift range, hyperpolarized ^129^Xe may be detected after inhalation in membrane tissues (M) and red blood cells (RBC) because of diffusion into alveolar septa from the airspaces; see Figure 7. RBC signals thereby provide information on blood volume, being probably the MRI method most specific to pulmonary capillary blood. It has been suggested that the RBC signal in established protocols of ^129^Xe dissolved‐phase imaging is reflective of capillary volume [91] and reductions reflective of micro‐thrombi, for example, in dyspneic patients after COVID infection [92]. However, it can generally also be affected by the rate of diffusion through the alveolar membrane and may thus not always be easily interpretable in terms of perfusion or blood volume. More recent work uses time‐resolved spectroscopy of the ^129^Xe dissolved phase to assess the pulsatility of the RBC signal which likely primarily probes periodic volume changes of blood in capillaries [93]. Also, an imaging method for assessment of RBC signal pulsatility has been proposed [94]. The ^129^Xe chemical shift saturation recovery (CSSR) spectroscopy method to be described in more detail later may also provide information on the capillary transit time of blood [95] but thorough validation of this parameter with established physiological measurements is pending.

Although being in an early stage of development, there is promise for the noninvasive assessment of different forms of pulmonary hypertension through assessment of RBC pulsations in time‐resolved spectroscopy [96, 97]. A limitation in the evaluation of perfusion using ^129^Xe MRI, also applying to other structural and functional measures, is the fact that poorly ventilated regions are difficult to assess, at least in common protocols with inhalation of a single dose.

### 3.5 Contrast‐Enhanced CT

Different approaches exist to create iodine maps and thereby probe lung perfusion in CT scans after injection of iodinated contrast agents. Dual‐energy CT employing two X‐ray sources with energies of different distances to iodine's K edge may be used to estimate the iodine content. The ability to acquire images only after contrast administration poses an advantage in terms of radiation dose [98]. A technically simpler technique for perfusion assessment showing advantages in terms of beam‐hardening artifacts is to subtract images before contrast enhancement from those after contrast enhancement, which generally requires image registration due to unavoidable motion [99]. The new generation of photon‐counting CTs also facilitates computation of iodine and virtual native images, which can be combined with assessment of ventilation by acquiring images in inspiration and expiration [63]; see Figure 5. Generally, photon‐counting CT requires less dose and has superior image quality compared to conventional energy‐integrating detector CT, which opens up the opportunity for functional lung CT in the clinic. Providing V and Q maps with much higher image resolution, functional photon‐counting lung CT has the potential to replace V/Q SPECT for the diagnosis of chronic thromboembolic pulmonary hypertension [100]. Using area‐detector CT, it is also possible to obtain dynamic contrast enhancement in axial scanning for quantitative perfusion assessment similar to MRI [101], albeit with limited volumetric coverage.

### 3.6 SPECT

Clinically, SPECT imaging after injection of radioactively labeled particles is still considered the gold standard for detection of chronic pulmonary embolism [59]. One of the advantages of SPECT imaging with regard to assessment of perfusion is the ability to simultaneously assess ventilation and thereby evaluate the nature of a perfusion defect with regard to V/Q matching. A further advantage may be the fact that perfusion in small vessels can be selectively imaged by choosing the size of injected particles to be in the range of 15–100 μm [59]. Again, the limited spatial resolution and use of ionizing radiation are disadvantages, although radiation doses are still lower compared to perfusion CT [102].

## 4 Alveolar Membrane Function

Gas exchange may be considered the primary function of the lung. Diseases affecting the function of the alveolar membrane by interstitial thickening, such as pulmonary fibrosis, or by pulmonary edema, such as COVID‐19, are associated with a significant mortality.

Clinically, the test of carbon monoxide uptake for determination of the diffusing capacity of the lung (DL_CO_) is well established and routinely applied. Reductions of diffusing capacity may be observed, for example, in the presence of emphysema or lung fibrosis. Apart from the fact that the measurement of diffusing capacity for the whole lung may have limited sensitivity for regional changes, the cause of reduced diffusing capacity may be difficult to assess from established lung function tests. While a decomposition of diffusing capacity into a membrane and capillary volume part is possible in principle, few pulmonologists acquire such data, and the underlying Roughton–Forster model may not be as generally valid as was thought for a long time [103].

### 4.1 ^129^Xe MRI

Imaging tests may address the aforementioned limitations of clinical lung function tests. Apart from oxygen‐enhanced MRI, which is able to measure diffusion in combination with ventilation and perfusion, ^129^Xe MRI is the only imaging method that can truly assess gas uptake with specificity to the individual contributions of tissue thickening, reduction of surface area, ventilation distribution, and perfusion.

#### 4.1.1 Dissolved‐Phase Imaging

As mentioned in the previous section, by virtue of xenon's good solubility in bodily fluids and ^129^Xe's large chemical shift range, hyperpolarized ^129^Xe MRI is able to track the fate of inhaled gas being taken up into the alveolar wall and transported away by the bloodstream. Compared to the established clinical test of carbon monoxide uptake, this may facilitate regional measurements of gas uptake efficiency, typically quantified as red blood cell/membrane ratio. It has also been proposed to model the clinically measured diffusing capacity by a decomposition into the contribution from membrane conductance and uptake into red blood cells and thereby derive this established quantity from ^129^Xe MRI [91].

Although imaging of the ^129^Xe dissolved phase was foreseen early [4], it is a relatively new technique and continues to evolve. Early work utilized the chemical shift displacement in the frequency‐encoding direction using Cartesian sampling to achieve a separation of signals from gas and dissolved phase [104]. Having the advantage of highly robust separation of phases, the method poses restrictions on minimum echo time and maximum receiver bandwidth. The latter also implies the disadvantage of a noticeable chemical shift artifact between red blood cells and membrane tissues, particularly in species like man with two clearly discernible dissolved‐phase resonances.

Later work used frequency‐selective excitation of the dissolved phase for suppression of the strong signal from gaseous ^129^Xe combined with radial trajectories. For best performance, RF pulses should be optimized for a given MRI system, for example, by varying their duration to account for distortions of the pulse shape created by the RF power amplifier, minimizing spurious excitation of gaseous ^129^Xe [105]. Methods for retrospective removal of artifacts from off‐resonant excitation have also been proposed [106]. Decomposition into signals originating from red blood cells and membrane tissues may then be achieved by methods similar to those used for water‐fat separation in ^1^H MRI [107, 108]. There is, however, no clear consensus yet whether single‐ or multi‐point Dixon techniques should be preferred.

Although dissolved‐phase imaging yields quantitative metrics after dividing signals through those from gas‐phase imaging, the ratios obtained are dependent on TR and flip angle [109] and do not take the temporal dynamics of gas uptake into account, thus showing a more qualitative picture of lung function. A further limitation is the still limited spatial resolution achievable both compared to ^129^Xe ventilation MRI and ^1^H MRI of the lung [110].

**FIGURE 8:**
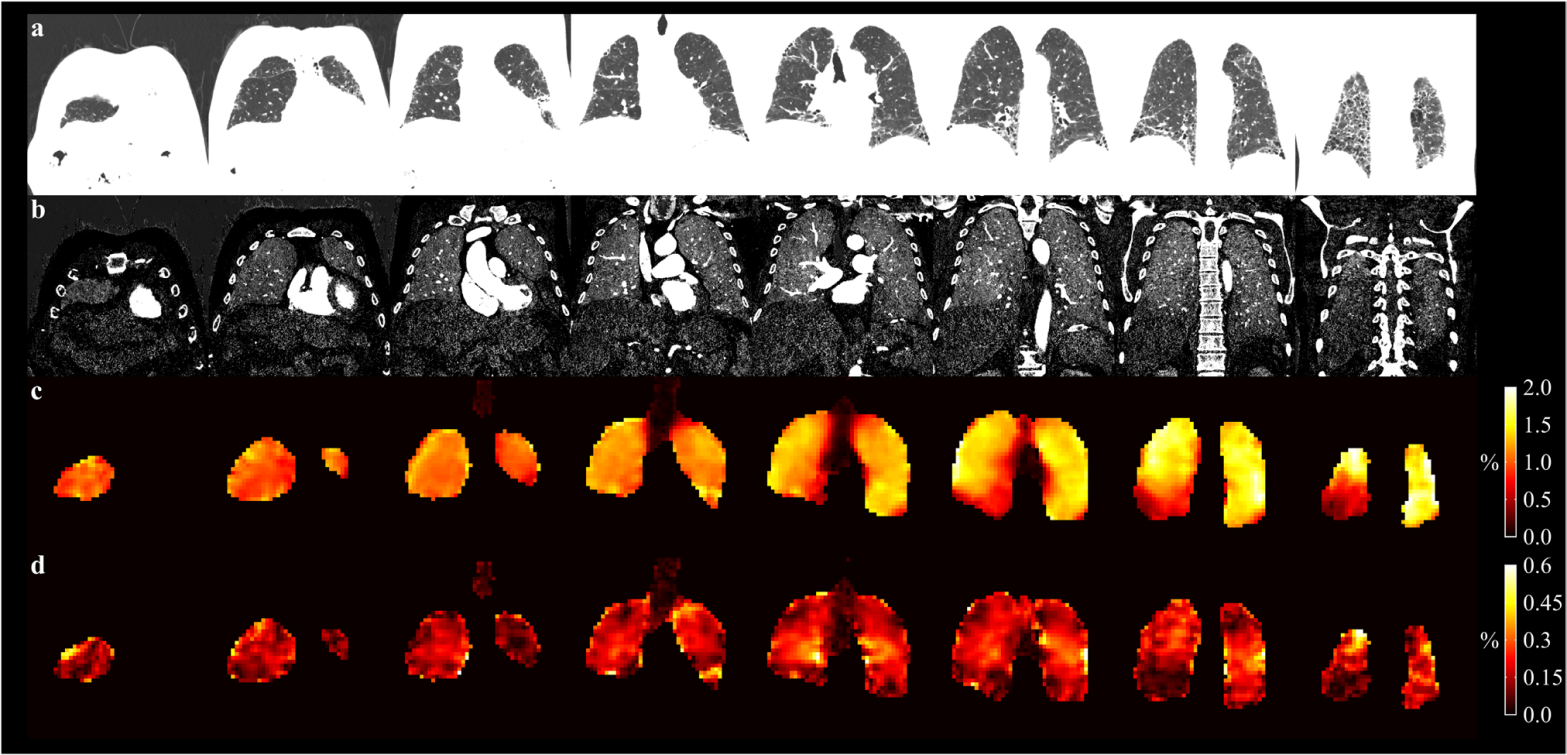
(a) Anatomical virtual native CT images and (b) iodine maps in similar slice location as ^129^Xe dissolved‐phase ratio maps for M‐Gas (c) and RBC‐Gas (d) in a 73‐year‐old male patient with idiopathic pulmonary fibrosis. Heterogeneity of gas uptake is observed in both ratio maps. RBC, red blood cell, M, membrane tissues.

Patients of diseases primarily affecting the lung's interstitium like pulmonary fibrosis may benefit strongly from the information gained through a ^129^Xe dissolved‐phase MRI scan since sensitivity both to disease and patterns of local disease stage have been suggested [111]; see Figure 8. The method is also sensitive to changes caused by COVID‐19 during the resolution phase [92, 112, 113].

Although specific signatures have also been observed in obstructive disease and in the presence of pulmonary nodules [104, 108], the clinical value of the method may be less clear in these cases. Consideration of normal age‐related changes in gas uptake for disease detection in ^129^Xe dissolved‐phase MRI is still a field of ongoing research despite recent progress [114]. Apart from diagnostic applications, the method may be a useful research tool for clinical studies employing models of lung inflammation, for example, for testing of drug efficacy [115].

#### 4.1.2 Chemical Shift Saturation Recovery

**FIGURE 9:**
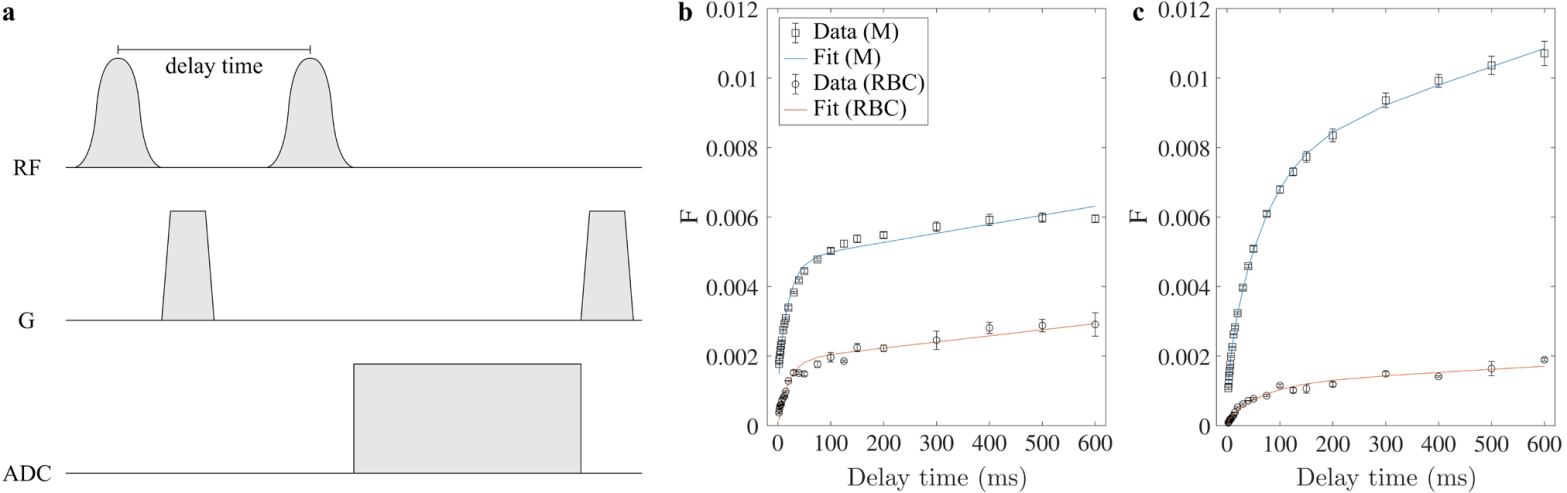
(a) ^129^Xe chemical shift saturation recovery (CSSR) sequence diagram and (b) uptake curves (ratio F of dissolved ^129^Xe over gaseous ^129^Xe) with model of xenon exchange (MOXE) fits in a healthy 29‐year‐old male volunteer and (c) a 73‐year‐old male patient with idiopathic pulmonary fibrosis. At short delay times, gas uptake is thought to be dominated by the lung surface–volume ratio. At intermediate delay times, uptake curves have a kink thought to be associated with the saturation of alveolar septa. Increased F at higher delay times is thought to be a consequence of blood flow. Increased septal thickness (15.4 μm vs. 8.1 μm) is apparent in the data from the fibrosis patient.

Chemical shift saturation recovery (CSSR) MR spectroscopy is an alternative approach for studying gas exchange and structure of alveolar septa. The sequence destroys the magnetization within the dissolved phase using saturation pulses and measures subsequent signal buildup, see Figure 9. For short delay times up to ~100 ms, the signal evolution may be approximated by diffusion within a one‐dimensional rod, showing the process of ^129^Xe filling the alveolar septa from the interfaces with the gas phase. The signal evolution at later times becomes dominated by the influence of blood flow. Various models have been developed for obtaining quantitative estimates of microstructural parameters like septal thickness, surface–volume ratio, and barrier thickness from CSSR data [95, 97, 116]. Validation of parameters obtained by comparison to histology has so far been limited to animal studies after the creation of clear pathology [117]. Based on numerical simulations, a limitation of these simplistic 1D models is that they do not account for the heterogeneity of lung microstructure, for example, in fibrotic lung disease [118].

Being originally developed as a spectroscopy sequence without spatially selective excitation, the method yielded no information on the regional distribution of the parameters. Various strategies have been developed in recent years to obtain regional information, either by use of multi‐channel coils with spatially varying sensitivity [119] or by addition of imaging gradients to the sequence with strategies for acceleration like a Look–Locker‐type acquisition [120]. Such a rapid acquisition also facilitates time‐resolved CSSR measurements, providing new insights into lung physiology [121].

The sensitivity of septal wall thickness derived from CSSR even to subclinical changes has been shown both by its age dependence [122] and its potential to discriminate apparently healthy smokers from healthy subjects [123]. A clinically relevant application could be the monitoring of patients suffering from pulmonary fibrosis [122].

## 5 Microstructure

Although imaging of the lung microstructure in the sense of mapping of microstructural parameters may not be functional imaging in a strict sense, it has strong implications for lung function in a variety of ways. A number of imaging tools are available to show various aspects of lung microstructure at the sub‐voxel length scale in the form of parameter maps.

### 5.1 Diffusion‐Weighted MRI of Inhaled Tracers

Gas transport within the lung is based both on convection and diffusion, with the diffusional component predominating in the smallest airways [124]. Paradoxically, while emphysema reduces the surface area of the lung, reducing its capacity for gas exchange, the destruction of alveolar walls improves diffusive gas transport in the lowest airway generations compared to the healthy lung, as evidenced by increased apparent diffusion coefficients (ADC) of inhaled tracer gases [125]. This is thought to provide a natural protection mechanism against the effects of mild emphysema [126].

#### 5.1.1 ADC Mapping

Technically, mapping of the ADC of inhaled tracer gases is typically performed using gradient‐echo based sequences employing a pair of bipolar gradients for diffusion sensitization. The fact that the magnetization of inhaled hyperpolarized gases diminishes throughout the measurement poses special requirements which make diffusion‐weighted spin‐echo sequences common in ^1^H MRI less useful. Further, the fact that diffusion‐weighted and unweighted acquisitions do not have equal starting magnetizations frequently makes a second unweighted acquisition necessary for compensation.

Due to the great differences between free diffusion coefficients of the common tracer gases ^3^He, ^129^Xe and fluorinated gases, ADCs obtained at equal diffusion time are sensitive to different aspects of lung microstructure [127]. In particular, the ADC obtained at short diffusion times and low free diffusion coefficients corresponding to short diffusive length scales can be expected to be related mainly to the surface–volume ratio of lung air spaces, while at higher length scales airway tortuosity is expected to come into play [127].

**FIGURE 10:**
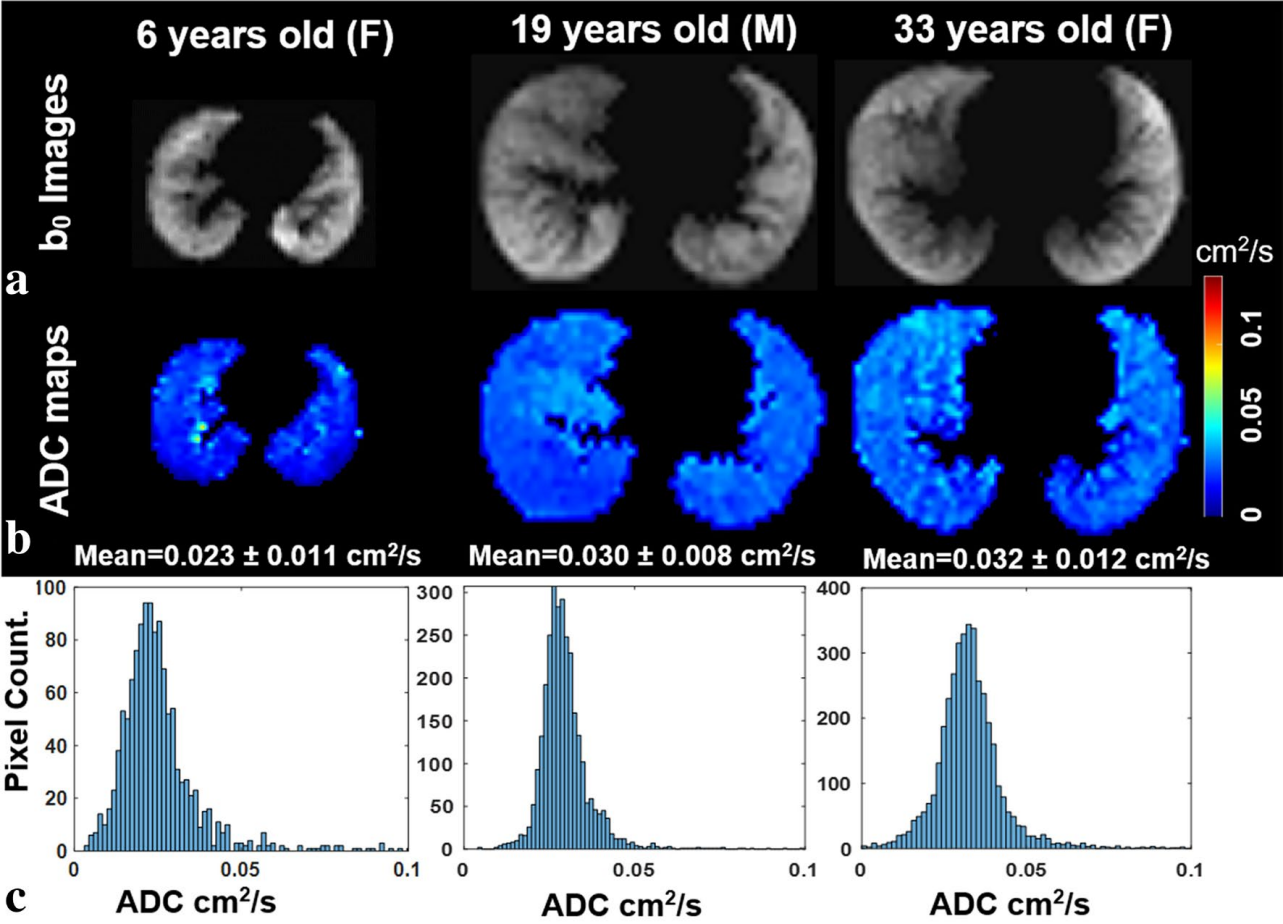
Hyperpolarized gas images with negligible diffusion weighting (b_0_) (a), apparent diffusion coefficient (ADC) maps (b), and ADC histograms (c) in a healthy child and two healthy adults. An increase in ^129^Xe ADC with age is apparent, suggesting an increase in airspace sizes in the growing lung. Reproduced with permission from [129]. F, female; M, male.

While emphysema detection could be a clinical application of ADC mapping of inhaled gases, CT is more widely available and the associated ionizing radiation is of less concern in patients typically of advanced age where emphysema could be suspected. The assessment of airspace enlargement in bronchopulmonary dysplasia may be a clinically relevant application [128]. Due to the strong correlation with surface–volume ratio—at least on a whole‐lung level—ADC measurements are an interesting method for assessing, for example, age‐related structural changes in vivo [129]; see Figure 10.

#### 5.1.2 Microstructure Quantification Using Multiple *b*‐Value Experiments

Quantitative estimates for structural dimensions of acinar airways may be obtained from gradient‐echo based diffusion‐weighted images using models such as the cylinder model developed by Yablonskiy et al. [130]. The non‐monoexponential decay of the MR signal of ^3^He or ^129^Xe in a multiple *b*‐value experiment is modeled by approximating airways as infinitely long cylinders, for which an equation describing the signal decay in terms of transverse and longitudinal diffusivities in closed form is known. Assuming a cylindrical airway geometry with alveolar sleeves, longitudinal and transverse diffusivities may be modeled in terms of microstructural parameters like the radius of acinar airways and alveolar sleeve depth [131].

The parameters may be used to obtain an estimate on mean chord length, also called mean linear intercept, which is a frequently used histological measurement. Excellent agreement between mean chord length estimated from ^3^He MRI and mean chord length from histology has been observed in a study with 6 human lung explants [132]. A limitation of the model is the assumption of a geometry representative of healthy human lung such that results obtained in disease may be difficult to interpret. It has also been suggested that airway branching in actual airways may affect the results [133]. Limitations with respect to potential clinical application are the high SNR requirement and relatively long acquisition time.

**FIGURE 11:**
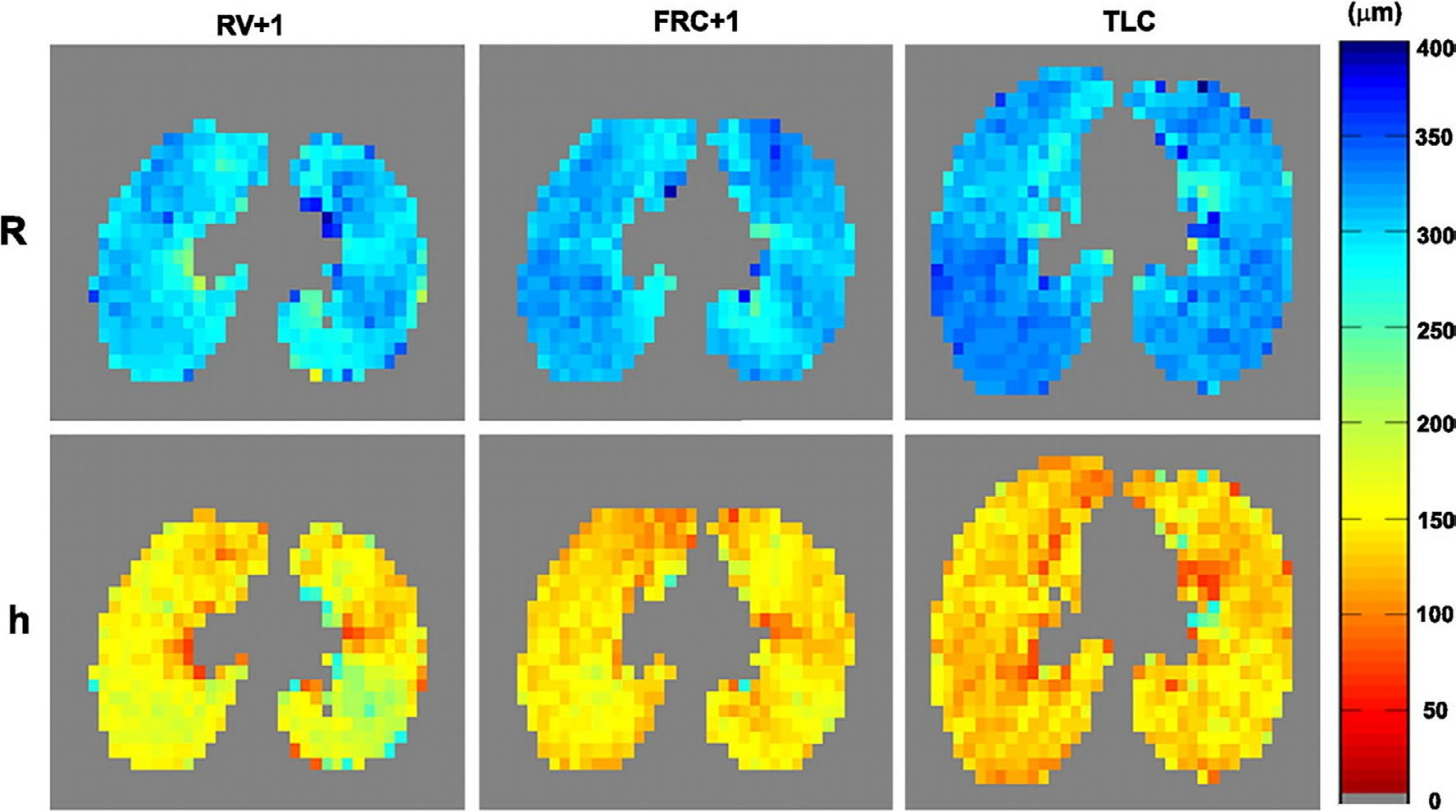
Alveolar duct radius *R* and alveolar sleeve depth *h* at multiple lung inflation levels (residual volume [RV] plus 1 L, functional residual capacity [FRC] plus 1 L and total lung capacity [TLC]) in a healthy volunteer. A reduction in the alveolar sleeve depth and an increase in the alveolar duct radius are observed. The relatively small magnitude of the increase in *R* may suggest alveolar recruitment as a relevant mechanism during respiration. Reproduced with permission from [134].

Consequently, the method may be more suitable to gain insights into lung physiology, for example, with respect to structural changes during respiration [134] or due to aging [135]; see Figure 11.

Another approach for deriving microstructural estimates from multiple *b*‐value experiments is based on the stretched exponential model. It is assumed that a voxel contains spins able to diffuse over different length scales during the experimental diffusion time Δ corresponding to a distribution of apparent diffusion coefficients *D*. By further assuming and fitting a stretched exponential signal decay *S*(*b*) = *S*_0_ exp (−(*b* DDC)^*α*^) with distributed diffusivity DDC and heterogeneity index *α*, the local distribution of diffusivities within a voxel may be obtained relating to a distribution of length scales *L*_D_ via the dependence *L*_D_ = (2Δ*D*)^1/2^. This allows the computation of an average length scale *L*_m,D_, which, however, is not directly interpretable in terms of standard histological quantities. It appears, however, to be consistently related with mean chord length estimated from the cylinder model in different diseases [136], although a differing relationship was observed at a different magnetic field strength [137]. The fact that no assumptions about the geometry of lung tissue are made may be an advantage over the cylinder model, although results may not be interpretable as easily.

As mentioned, the stretched exponential model has been applied in a range of diseases including COPD, asthma, and idiopathic pulmonary fibrosis [136]. It may be helpful in the characterization of patients, for example, for clinical studies or in studies with disease models in preclinical imaging [138].

#### 5.1.3 Microstructure Quantification Using Multiple Diffusion Time Experiments

**FIGURE 12:**
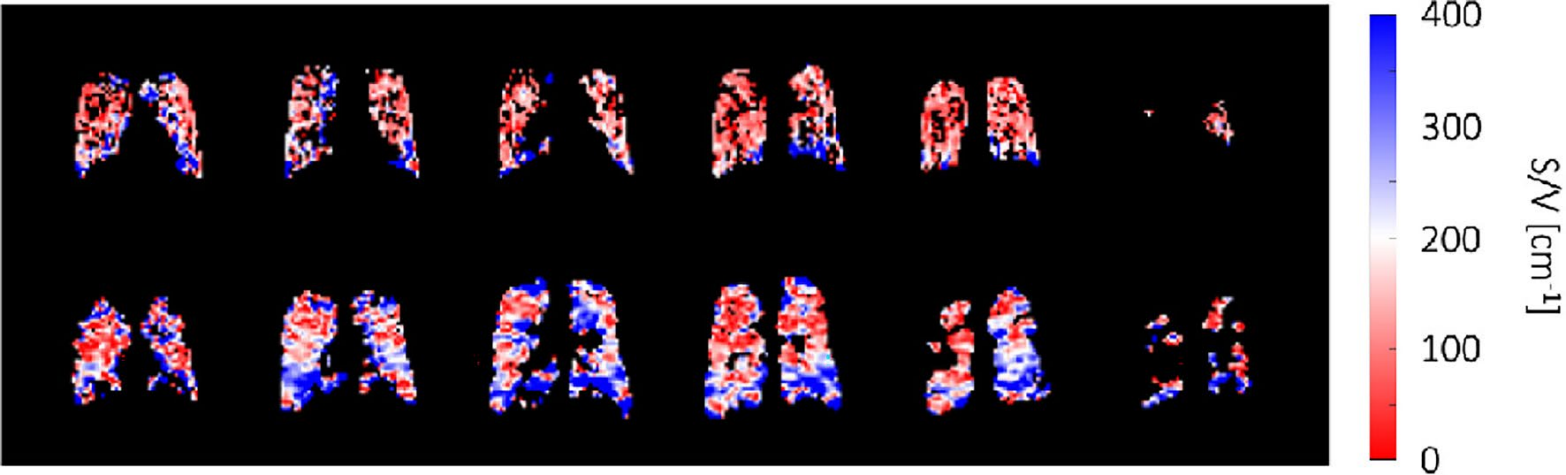
Measurement of the lung surface–volume (S/V) ratio using ^19^F (top row) and ^129^Xe (bottom row) diffusion‐weighted MR imaging at variable diffusion time in a patient with chronic obstructive pulmonary disease, forced expiratory volume in 1 s 63% of predicted value. Reproduced from [141], published under the terms of a Creative Commons Attribution License, https://creativecommons.org/licenses/by/4.0/.

As previously mentioned, the ADC of inhaled tracer gases at very short diffusive length scales is expected to depend linearly on the surface–volume ratio of lung airspaces. This is thought to apply to fluorinated gas MRI quite generally and to ^129^Xe MRI at sub‐millisecond diffusion times [127, 139]. Achievement of low enough diffusion times is technically challenging, especially at high surface–volume ratios; however, such that the introduction of higher‐order terms in the analysis of experiments involving ^129^Xe MRI at multiple diffusion times for surface–volume estimation may be necessary [140]. A relatively good agreement between surface–volume ratio obtained from ^129^Xe MRI and ^19^F MRI has been observed in patients of COPD [141]; see Figure 12. The fact that no assumptions on the underlying airway geometry are made may be an advantage of this method over the more established cylinder model applied to multiple *b*‐value experiments.

### 5.2 UTE MRI

**FIGURE 13:**
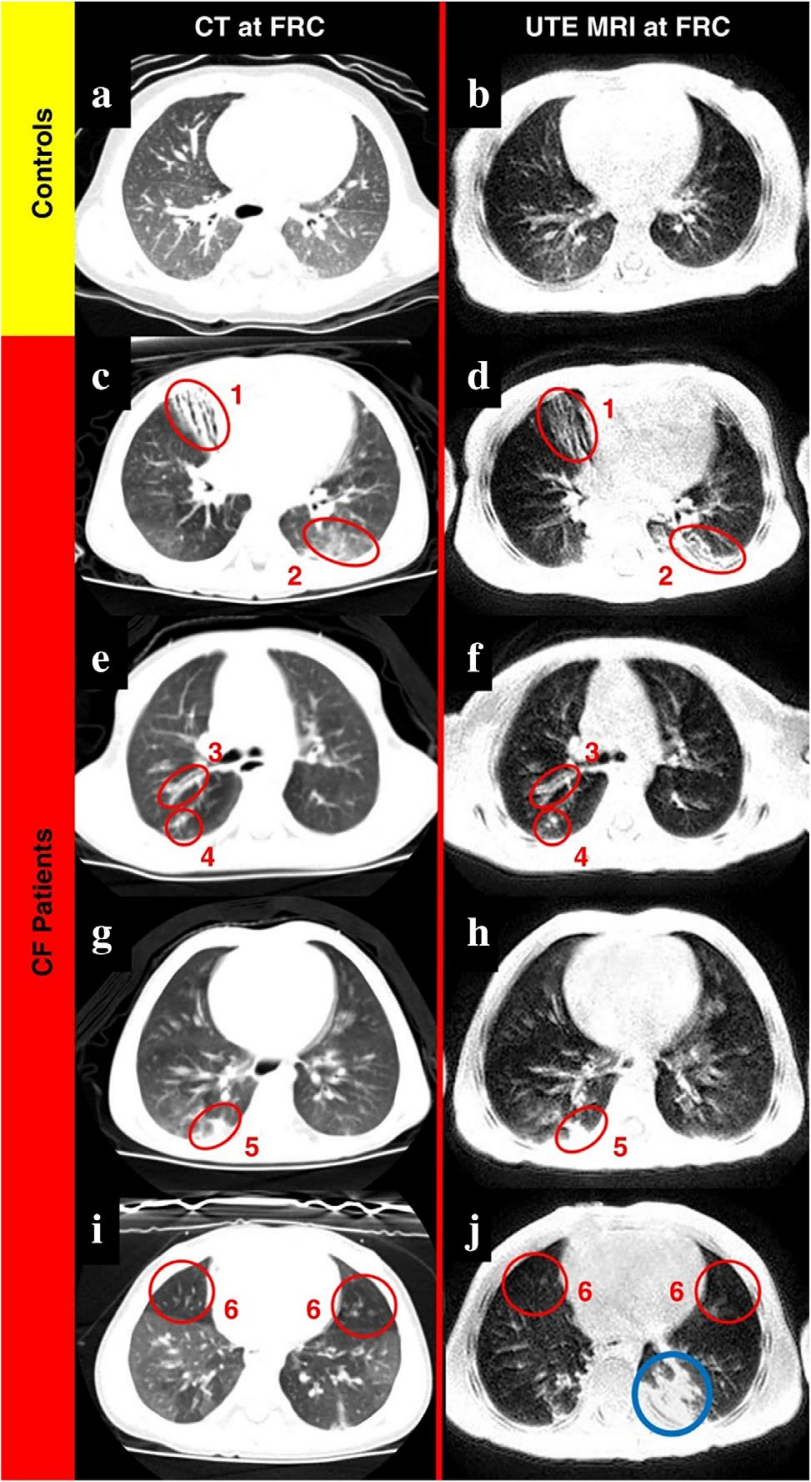
Ultra‐short echo time (UTE) MRI and CT imaging in a healthy control and patients with cystic fibrosis (CF) obtained at functional residual capacity (FRC). Most features apparent on CT can also be observed in UTE MRI. Reprinted with permission of the American Thoracic Society [142]. Copyright © 2016 American Thoracic Society. All rights reserved. Annals of the American Thoracic Society is an official journal of the American Thoracic Society.

Given the short transverse relaxation times of lung tissue at clinical field strengths as a consequence of the numerous air‐tissue interfaces and associated susceptibility jumps, great improvements of signal intensity in ^1^H MRI of the lung can be achieved by going to shorter echo times. Images acquired at ultra‐short echo time (UTE) are typically obtained in the form of 3D volumes and quality may come close to those of structural images obtained from CT despite still impaired resolution; see Figure 13. Apparent transverse relaxation rates measured by multi‐echo UTE are sensitive to tissue destruction and are able to differentiate disease severity in COPD [143].

A natural clinical application of UTE MRI for structural assessment could be longitudinal monitoring of bronchopulmonary dysplasia or cystic fibrosis lung disease where, for example, mucus plugging, bronchiectasis, or ground‐glass opacities may be detected [142].

### 5.3 CT

CT imaging produces high‐resolution images of the lung in exquisite detail compared to other medical imaging modalities but still shows the lung parenchyma as a homogeneous tissue without resolving its microstructure. Yet, given the fact that the average thickness of alveolar septa is only weakly changed in a number of lung diseases, there may be a strong correlation between radiodensity on CT in Hounsfield units and the surface–volume ratio as evidenced in previous studies comparing imaging results with histology in emphysema [144]. Further, the dimensions of airways resolvable with CT were found to be predictive for small airway dimensions below the resolution limit [145]. More qualitatively, the extent of emphysema may be quantified by the fraction of lung tissue with density below −950 HU in inspiration [62].

### 5.4 Dark‐Field X‐Ray Imaging

The introduction of grating interferometers to the field of X‐ray dark‐field imaging has facilitated the construction of dark‐field imaging devices using conventional X‐ray tubes, thus being associated with low technical complexity and potentially low cost in a future commercial setting. Being sensitive to fluctuations of tissue density on a nanometer to micrometer scale, the imaging method shows strong contrast between, for example, lung tissue and bones compared to surrounding tissues.

**FIGURE 14:**
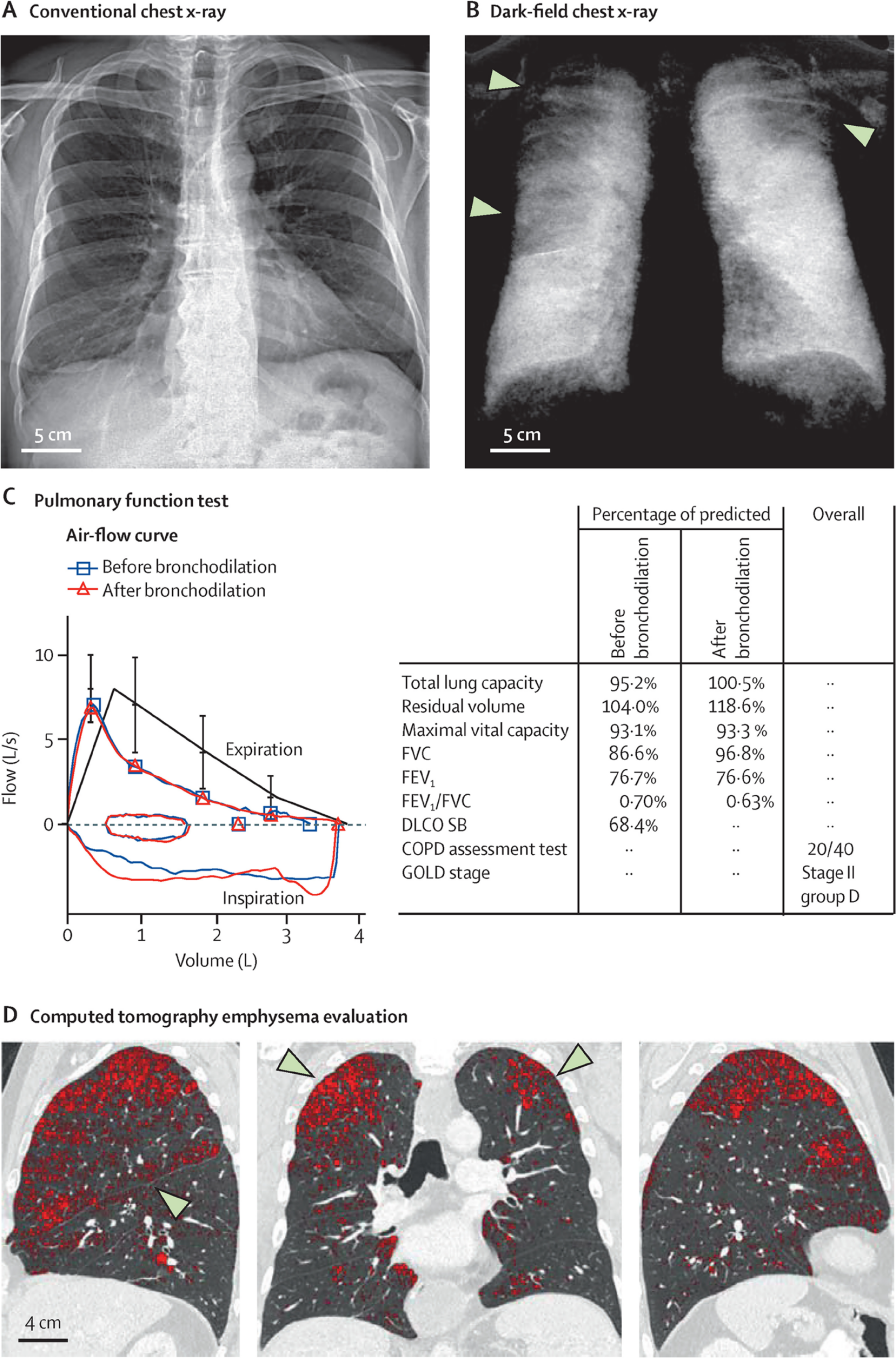
Conventional chest X‐ray and dark‐field X‐ray image (a/b) in a subject with chronic obstructive pulmonary disease with results from spirometry (c) and CT images (d). Emphysematous regions are readily apparent in the dark‐field X‐ray image but hard to see on conventional chest X‐ray. Reproduced from [146], published under the terms of a Creative Commons Attribution License, https://creativecommons.org/licenses/by/4.0/. COPD, chronic obstructive pulmonary disease; DLCO SB, diffusing capacity of the lung for carbon monoxide measured in a single breath; FEV1, forced expiratory volume in 1 s; FVC, forced vital capacity; GOLD, global initiative for chronic obstructive lung disease.

An important potential clinical application of this technique could be the detection of emphysema [146], given the potentially improved sensitivity over chest X‐ray and reduced radiation dose compared to CT; see Figure 14. Yet, reduced signal in dark‐field imaging of the chest is not specific to emphysema as it may also be caused by fibrosis, for instance [147]. Recently, it has been shown that dark‐field signal intensity is affected by lung inflation level with maybe somewhat unexpectedly increasing signal at higher inflation level post‐mortem [148]. This was explained by an opening of additional alveoli at higher inflation levels although the surface–volume ratio is generally expected to decrease at increased lung inflation. More work seems necessary to obtain a thorough understanding of dark‐field signal intensity in terms of established histological quantities. Current developments will enable also clinical dark‐field CT studies with human patients in the near future.

## 6 Summary and Outlook

Great advances have been made to develop and translate quantitative functional lung imaging tools to improve diagnosis, monitoring, and prognostication of patients with lung disease. A great variety of methods exists for functional imaging of the lung, each with its own strengths and weaknesses. While some methods are well‐established clinically and well understood in terms of the underlying contrast mechanisms, others have so far not left the experimental stage and may require more validation before routine application is possible.

In particular, there appears to be a number of experimental MRI methods with great potential for clinical application. Progress in this regard is likely associated with commercialization by companies which take the efforts to develop methods technically ready for the clinic and solve regulatory issues. As an example, the recent approval of hyperpolarized ^129^Xe for imaging of ventilation by the United States Food and Drug Administration is an important step in the right direction, although a number of issues like the regulatory situation in other countries and the technical complexity remain.

The need for functional pulmonary imaging both for clinical and clinical research applications is likely to increase in the decades to come as a result of the increasing prevalence of lung diseases and the associated urgent need for the development of novel therapies. From a scientific perspective, many aspects of lung function are still to be elucidated, and medical imaging has most likely an important role to play here due to its noninvasive nature and methodological versatility. Consequently, the future of the field of functional pulmonary imaging is bright!
